# Diversity Dynamics in Nymphalidae Butterflies: Effect of Phylogenetic Uncertainty on Diversification Rate Shift Estimates

**DOI:** 10.1371/journal.pone.0120928

**Published:** 2015-04-01

**Authors:** Carlos Peña, Marianne Espeland

**Affiliations:** 1 Laboratory of Genetics, Department of Biology, University of Turku, Turku, Finland; 2 Museum of Comparative Zoology, Harvard University, Cambridge, USA; 3 Department of Organismic and Evolutionary Biology, Harvard University, Cambridge, USA; Oxford Brookes University, UNITED KINGDOM

## Abstract

The species rich butterfly family Nymphalidae has been used to study evolutionary interactions between plants and insects. Theories of insect-hostplant dynamics predict accelerated diversification due to key innovations. In evolutionary biology, analysis of maximum credibility trees in the software MEDUSA (modelling evolutionary diversity using stepwise AIC) is a popular method for estimation of shifts in diversification rates. We investigated whether phylogenetic uncertainty can produce different results by extending the method across a random sample of trees from the posterior distribution of a Bayesian run. Using the MultiMEDUSA approach, we found that phylogenetic uncertainty greatly affects diversification rate estimates. Different trees produced diversification rates ranging from high values to almost zero for the same clade, and both significant rate increase and decrease in some clades. Only four out of 18 significant shifts found on the maximum clade credibility tree were consistent across most of the sampled trees. Among these, we found accelerated diversification for Ithomiini butterflies. We used the binary speciation and extinction model (BiSSE) and found that a hostplant shift to Solanaceae is correlated with increased net diversification rates in Ithomiini, congruent with the diffuse cospeciation hypothesis. Our results show that taking phylogenetic uncertainty into account when estimating net diversification rate shifts is of great importance, as very different results can be obtained when using the maximum clade credibility tree and other trees from the posterior distribution.

## Introduction

Hostplant shifts have been invoked to be responsible for a great part of the biodiversity of herbivorous insects [1]. The study of the evolution of hostplant use has spawned several theories explaining the evolutionary interactions between plants and insects [2]: the “escape-and-radiate” hypothesis [3], the “oscillation hypothesis” [4, 5] or “diffuse cospeciation” [2] and the “musical chairs hypothesis” [6].

The butterfly family Nymphalidae has been an important taxon for developing some of the mentioned hypotheses. Nymphalidae contains around 6000 species [7], and several members are considered model organisms in evolutionary biology [8–10]. The family most likely originated around 94 MYA in the mid Cretaceous. Diversification of the group began in the Late Cretaceous and most major radiations (current subfamilies) appeared shortly after the Cretaceous-Paleogene (K-Pg) boundary [11]. Several studies have used time-calibrated phylogenies and diversification models to reconstruct the evolutionary history of the group to identify patterns of accelerated or decelerated diversification of some Nymphalidae clades [11–14]. For example, it has been suggested that climate change in the Oligocene and the subsequent diversification of grasses has led to diversification of the subfamily Satyrinae [15] due to the abundance of grasses over extensive geographic areas (“resource abundance-dependent diversity dynamics” hypothesis): Fordyce (2010) [13] found increased net diversification rates in some Nymphalidae lineages after a major hostplant shift, which appears to be in agreement with the “escape-and-radiate” model of diversification [3].

Although it has been suggested that part of the great diversity of Nymphalidae butterflies is a result of hostplant-insect dynamics, it is necessary to use modern techniques to investigate whether the diversification patterns of Nymphalidae are in agreement with the theoretical predictions. It is necessary to test whether the overall diversification pattern of Nymphalidae is congruent with events of sudden diversification bursts due to hostplant shift or climatic events [5, 16].

In this study, we used a time-calibrated genus-level phylogenetic hypothesis for Nymphalidae butterflies [14] to investigate patterns of diversification. We applied the statistical method MEDUSA [17, 18], to study the diversification pattern of Nymphalidae butterflies. MEDUSA fits likelihood models of diversification into a time-calibrated tree and tests whether allowing increases or decreases in speciation and extinction rates within the tree produces better fit of the models. MEDUSA is able to take into account unsampled extant species diversity during model fitting and it is normally applied to the maximum clade credibility phylogenetic tree. Particularly, we wanted to study the effects of phylogenetic uncertainty and by using the extended MEDUSA method called MultiMEDUSA [17]. We also tested whether hostplant association dynamics can explain the diversification patterns of component Nymphalidae lineages by testing whether character states of hostplant use affected the diversification pattern of those lineages employing the binary speciation and extinction model (BiSSE) as implemented in the R package diversitree [19].

## Materials and Methods

### Data

**Fig 1:**
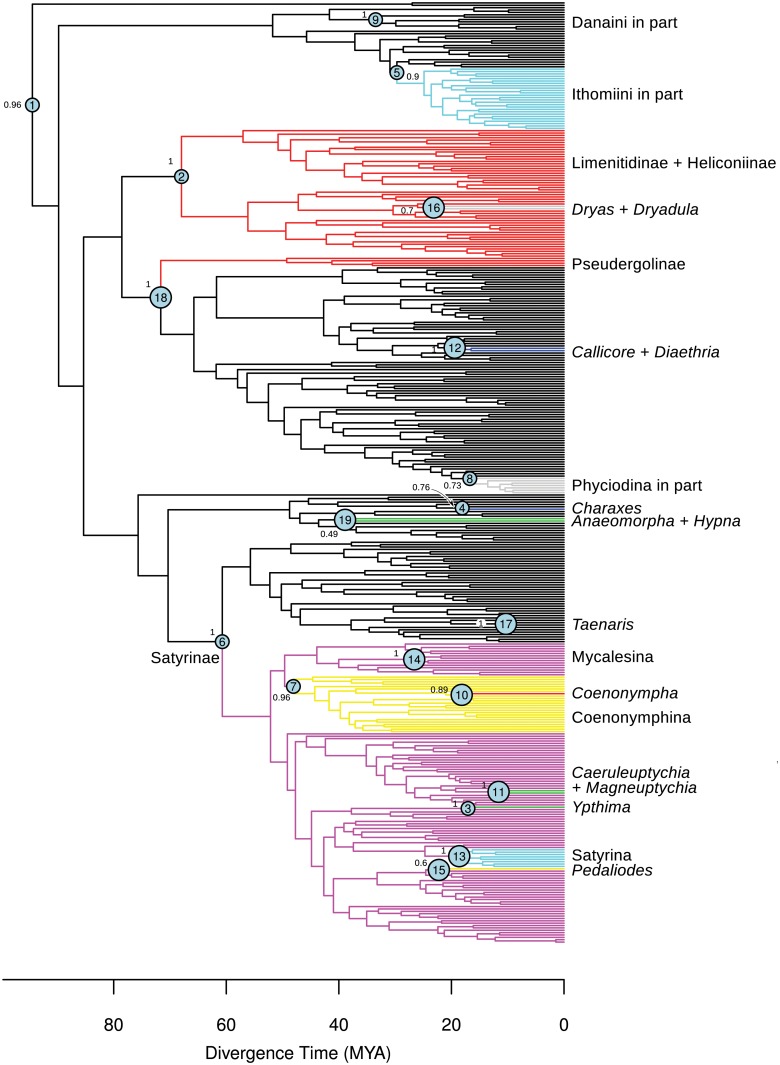
Results of the MEDUSA analysis run on the maximum clade credibility tree from Wahlberg et al. (2009). Rate shifts were estimated for the following nodes (besides the background rate): 1) root, 2) Limenitidinae + Heliconiinae, 3) *Ypthima*, 4) *Charaxes*, 5) Ithomiini in part, 6) Satyrinae, 7) Coenonymphina 8) Phyciodina in part, 9) Danaini in part, 10) *Coenonympha*, 11) *Caeruleuptychia* + *Magneuptychia*, 12) *Callicore* + *Diaethria*, 13) Satyrina, 14) Mycalesina, 15) *Pedaliodes*, 16) *Dryas* + *Dryadula*, 17) *Taenaris*, 18) Pseudergolinae, 19) *Anaeomorpha* + *Hypna*. Circles on nodes indicate the diversification shift number as found by MEDUSA. Numbers next to circles indicate the posterior probability values for such nodes.

For analyses, we used the phylogenetic trees from the study of Wahlberg et al. (2009) [14] that were generated using DNA sequence data from 10 gene regions for 398 of the 540 valid genera in Nymphalidae. We employed the maximum clade credibility tree (MCC tree) (Fig 1) as well as a random sample of 1000 trees from their BEAST run after burnin [14]. Their original BEAST run was for 40 million generations. We used a burnin of 25 million generations and took a random sample of 1000 trees using Burntrees v.0.1.9 http://www.abc.se/~nylander/ (S1 File) in order to account for phylogenetic uncertainty when performing the diversification analyses.

Species richness data for Nymphalidae genera were compiled from several sources including the specialist-curated lists [20, 21] and curated lists of the Global Butterfly Names project [22]. We assigned the species numbers of genera not included in the phylogeny to the closest related genus that was included in Wahlberg et al. (2009) [14] study according to available phylogenetic studies [23–34], taxonomical classification and morphological resemblance when no phylogenies were available (S1 Dataset).

Hostplant data for Nymphalidae species were compiled from several sources including [35], HOSTS database (http://bit.ly/YI7nwW), [36–38] and others (S2 and S3 Dataset) for a total of 6586 hostplant records, including 428 Nymphalidae genera and 143 plant families and 1070 plant genera. It was not possible to find any hostplant data for 35 butterfly genera.

### Analyses of Diversification

We used the statistical software R version 3.0.1 [39] in combination with the APE [40], GEIGER [41], MEDUSA [17] and diversitree [19] packages along with our own scripts to perform the analyses (included as S2 and S3 File). All analyses were run on the 1000 random trees from Wahlberg et al. (2009) [14], on the maximum clade credibility tree derived from these and the MCC tree [14].

### Detecting diversification shifts on phylogenetic trees

Patterns of diversification in Nymphalidae were analyzed by using MEDUSA version 093.4.33 [17] on the maximum clade credibility tree from Wahlberg et al. (2009) [14]. MEDUSA fits alternative birth-death likelihood models to a phylogenetic tree in order to estimate changes in net diversification rates along branches. MEDUSA estimates likelihood and AIC scores for the simplest birth-death model, with two parameters, the rates r: net diversification = speciation (b) − extinction (d) and (*ɛ*: relative extinction = d/b. The maximum-likelihood values are recalculated when each branch in the tree is selected to contain an additional breakpoint (additional parameters b and d) in net diversification rate. The process is repeated by fitting additional breaking points to the tree. The fit of alternative models is compared using AIC scores and stop when the improvement of the incrementally more complex models is smaller than a cutoff value which depends of the number of terminals. MEDUSA finds the likelihood of the models after taking into account branch lengths and number of species per lineage [17]. Most studies using MEDUSA only run the method on a single tree, usually the maximum clade credibility tree, which makes the assumption that this tree is correct. We wanted to study the effects of phylogenetic uncertainty on estimation of net diversification rate shifts and therefore selected 1000 random genus-level trees from the posterior distribution of the Bayesian run from Wahlberg et al., 2009 [14] (MultiMEDUSA, S1 File). We calculated a new MCC tree from the selection of trees, ran MEDUSA and MultiMEDUSA on the selection of 1000 trees and summarized the estimated changes in net diversification rates for nodes across all trees. Patterns of change in net diversification rates are considered significant if they are found at the same node in at least 90% of the trees. We also expected to find similar values of net diversification (r = b − d) and relative extinction rate (*ɛ* = d/b) values across the 1000 trees for the nodes where changes in diversification tempo occurs. We used the AICc threshold of 7.8 units, as estimated by MEDUSA, as the limit for a significantly better fit to select among increasingly complex alternative models.

### Estimation of trait-dependent speciation rates

As the MEDUSA and MultiMEDUSA approaches estimated an increase in net diversification in the clade Ithomiini, we tested whether this pattern can be explained by an increase in the birth-rate due to hostplant use and performed analyses using the BiSSE model [42] as implemented in the R package diversitree [19]. The Markov Chain Monte Carlo algorithm was run for 10000 generations discarding the first 7500 as burnin. Because most of Nymphalidae butterflies are restricted to use one plant family as hostplant (232 genera use only one plant family versus 176 that use more than one; based on our data in S2 Dataset), the character states can be coded as presence/absence, for which the BiSSE analysis is better suited. BiSSE was designed to test whether a binary character state has had any effect on increased net diversification rate for a clade [42]. We used our compiled data of hostplant use to produce a binary dataset for the character “feeding on the plant family Solanaceae” with two states (absence = 0; presence = 1) (S4 Dataset). The family Solanaceae include the hostplants of the diverse Ithomiini butterflies and closest relatives [9]. We analyzed the data using BiSSE employing the Markov Chain Monte Carlo algorithm on the maximum clade credibility tree, taking into account missing taxa by using the parameter “sampling factor” (sampling.f) in diversitree. We also run a BiSSE analysis to test whether the trait “feeding on Apocynaceae” had any effect on net diversification rates found for Nymphalidae lineages feeding on Apocynaceae. We also used likelihood ratio tests to evaluate the influence of Solanaceae-feeding on diversification rates by comparing a constrained versus a relaxed model of diversification. The constrained model assumes no effect of hostplant use on diversification by enforcing equal speciation rates across both character states. In the relaxed model, all speciation and extinction parameters are estimated for each character state. The analyses were run across a sample of 250 trees from the selected 1000 random trees from the posterior distribution. The records of *Vanessa* and *Hypanartia* feeding on Solanaceae [37, 43] might be incorrect as it is unlikely that these species are able to feed on members of this plant family. We tested whether coding these two genera as not feeding on Solanaceae affected our results. We expected that coding these two genera as either absence or presence would not have a significant effect on our results. It has been shown that BiSSE performs poorly under certain conditions [44]. However, our data has adequate number of taxa under analysis (more than 300 tips), adequate speciation bias (between 1.5× and 2.0×), character state bias (around 8×) and extinction bias (around 4×) for the analysis of Solanaceae hostplants. Thus, BiSSE is expected to produce robust results [44].

## Results

### Detecting diversification shifts on the maximum clade credibility tree

The MEDUSA analysis on the MCC tree in combination with richness data estimated 18 significant changes in the tempo of diversification in Nymphalidae history (Fig 1; Table 1). The corrected AICc acceptance threshold for adding subsequent piecewise birth-death processes to the overall model was set to 7.8 units, as prescribed by MEDUSA. In all MEDUSA analyses, the maximum number of inferred diversification shifts in all trees was 26. The background net diversification rate for Nymphalidae was estimated as r = 0.092 lineages per Million of years and the AICc score for the best fit model was 5090.5 (Table 1). MEDUSA also estimated that the basic constant birth-death model was not a better explanation for our data (AICc = 5449.3).

**Table 1:** Significant net diversification rate shifts found in the MEDUSA analysis of the Nymphalid maximum clade credibility tree.

Shift N°	Shift.Node	Model	r	LnLik.part	AICc	Taxa
1	399	yule	0.092459	-1055.957		Nymphalidae (root)
2	691	bd	0.054129	-406.3703		Limenitidinae + Heliconiinae
3	299	yule	0.311199	-6.3058		*Ypthima*
4	224	yule	0.290989	-6.2601		*Charaxes*
5	750	yule	0.186913	-147.4146		Oleriina + Ithomiina + Napeogenina + Dircennina + Godyrina
6	405	yule	0.116252	-555.0276		Satyrinae
7	495	yule	0.064656	-124.9143		Coenonymphina
8	609	yule	0.240562	-35.0908		Phyciodina in part
9	787	bd	0.042332	-43.7819		Danaini in part
10	231	yule	0.209416	-4.7955		*Coenonympha*
11	478	yule	0.311684	-9.2218		*Caeruleuptychia* + *Magneuptychia*
12	659	yule	0.219253	-11.2686		*Callicore* + *Diaethria*
13	444	yule	0.220615	-43.7812		Satyrina
14	524	yule	0.190754	-26.0651		Mycalesina
15	355	yule	0.234041	-6.2013		*Pedaliodes*
16	714	yule	0	0		*Dryas* + *Dryadula*
17	377	yule	0.311671	-4.1986		*Taenaris*
18	688	yule	0.024724	-17.5256		Pseudergolinae
19	583	yule	0	0	5090.492	*Anaeomorpha* + *Hypna*

Shift.Node = node number, Model = preferred diversification model by MEDUSA, r = net diversification rate, LnLik.part = log likelihood value.

Some of the 18 changes in diversification correspond to rate increases in very species-rich genera: *Ypthima* (r = 0.311), *Charaxes* (r = 0.291), *Callicore* + *Diaethria* (r = 0.220), *Pedaliodes* (r = 0.196) and *Taenaris* (r = 0.234). We found rate increases for other clades as well such as: Mycalesina (r = 0.191), Oleriina + Ithomiina + Napeogenina + Dircennina + Godyridina (r = 0.187), Satyrinae (r = 0.116), Phyciodina in part (r = 0.241) and Satyrina (r = 0.221), *Coenonympha* (r = 0.209), *Caeruleuptychia* + *Magneuptychia* (r = 0.312) and *Taenaris* (r = 0.312). We also found decreases in net diversification rates for Limenitidinae + Heliconiinae (r = 0.0541), part of Danaini (r = 0.0423), Pseudergolinae (r = 0.024) and Coenonymphina (r = 0.065) (Table 1).

### Phylogenetic uncertainty in the MultiMEDUSA approach

We used MEDUSA to find out whether taking into account the phylogenetic signal from the random sample of 1000 trees from the posterior distribution can return similar estimates of diversification to the values obtained from the MCC tree. We ran MultiMEDUSA on the random sample of 1000 trees (S1 File) from the posterior distribution and compared the results with a MEDUSA analysis on the MCC tree derived from this sample (S2 Supporting Information).

We found that the analysis by MultiMEDUSA on the 1000 trees estimated lower median net diversification rates for the diversification shifts found by MEDUSA on the MCC tree derived from the random sample of trees (Table 2). Although the diversification pattern found by MEDUSA and MultiMEDUSA was the same, the latter consistently estimated lower rates. Furthermore, the shifts recovered with low net diversification rate on the MCC were recovered with negative net diversification rate by MultiMEDUSA. The background diversification and all shifts found by MEDUSA on the 1000 trees are provided as an R object in S4 File.

**Table 2:** Differences in rates estimated by MEDUSA on the MCC tree from the sample of trees from the posterior distribution and the MultiMEDUSA approach. Shift consistently recovered across the sample of trees in bold face.

Shift.Node	rate by MEDUSA	Median rate by MultiMEDUSA	probability of being recovered
1	0.092	not found	0.000
2	0.055	-0.030	0.864
**3**	0.184	**0.219**	**0.961**
**4**	0.166	**0.212**	**0.996**
**5**	0.111	**0.101**	**0.927**
6	0.119	0.039	0.131
7	0.066	-0.052	0.195
8	0.232	0.166	0.319
9	0.042	-0.049	0.897
10	0.058	0.149	0.619
11	0.311	0.208	0.831
**12**	0.219	**0.135**	**0.911**
13	0.082	0.117	0.276
14	0.099	0.115	0.379
15	0.113	0.127	0.825
16	0.222	-0.005	0.659
17	0.243	0.248	0.785
18	0.192	-0.064	0.024
19	0.064	-0.087	0.381

We also compared the results from MultiMEDUSA (derived from the sample of 1000 trees) with the shifts found by MEDUSA on the MCC tree derived from this random sample. In the summary statistics, MultiMEDUSA reports the frequency of the diversification shifts found in the trees (parameter sum.prop). Thus, if a node is found in only half of the 1000 trees, but the phylogenetic signal was strong enough to be picked up by MEDUSA and a node shift was found most of the time, then the sum.prop should be close to 1. For example, the Charaxes + Polyura clade was found in only 256 trees, however MultiMEDUSA was consistently able to find a diversification shift for that node and the sum.prop value is 0.996.

For the diversification shifts found in both the MCC tree and most of the samples of 1000 trees (frequency more than 90%; Table 2), the MultiMEDUSA approach recovered different rates of diversification than those found when the MCC tree alone was used.

There were four net diversification rate increases found in the trees from the random sample (Table 2): (i) the genus *Ypthima* (*r* = 0.22); (ii) the genus *Charaxes* (*r* = 0.21); (iii) Ithomiini subtribes Oleriina + Ithomiina + Napeogenina + Dircennina + Godyridina (*r* = 0.10); and (iv) the clade of *Callicore* + *Diaethria* (*r* = 0.135).

**Fig 2:**
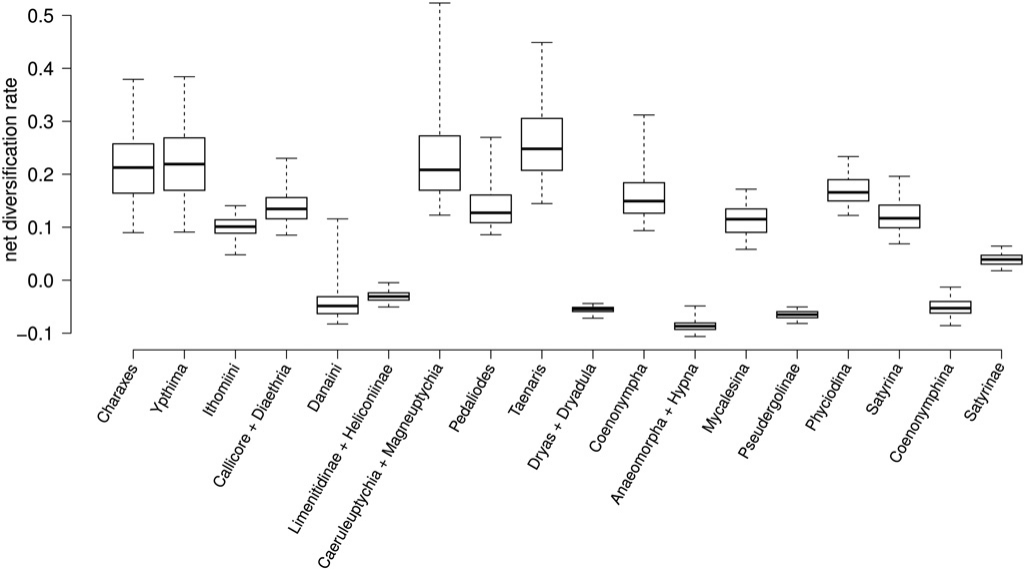
Diversification rates for taxa estimated by MultiMEDUSA on the samples of 1000 random trees.

MultiMEDUSA provided mean and standard deviation statistics for the diversification values on the shifts on the 1000 trees (S6 Fig and S1 Text), and found that the estimated net diversification rate values had great variation across the posterior distribution of trees. A boxplot of the net diversification rate values estimated for the clades that appear in the MCC tree shows that some shifts are estimated as increased or slowed diversification pace depending on the tree used for analysis (Fig 2). This variation is especially wide for the clade formed by the genera *Magneuptychia* and *Caeruleuptychia* because MEDUSA estimated diversification values from six times the background net diversification rate (r = 0.5234) to almost zero (r = 1.22e-01). The rates for *Taenaris* were between 0.14 and 0.44 (mean value 0.25). Similar degrees of variation were found in the nodes for *Ypthima*, *Charaxes* and *Coenonympha* (Fig 2). The net diversification rates estimates for the clades (Oleriina + Ithomiina + Napeogenina + Dircennina + Godyridina), Limenitidinae + Heliconiinae and Pseudergolinae are relatively consistent across the 1000 trees (Fig 2).

**Fig 3:**
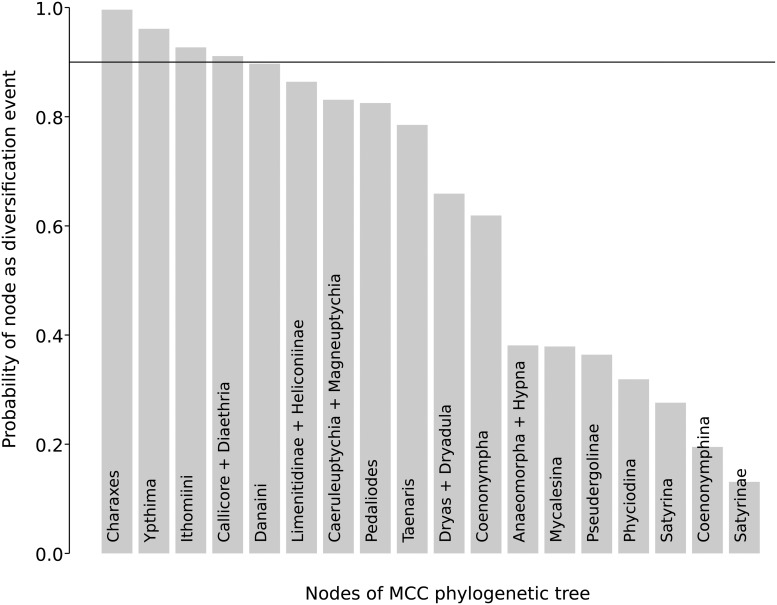
Results of MultiMEDUSA analysis showing the probability of specific nodes being characterized by significant shifts in diversification rate.

It is also evident that not all the diversification shifts estimated on the MCC tree are consistently recovered in most of the 1000 trees. Some of the shifts in the MCC tree are recovered in very few trees, for example the shift for the clade Satyrinae is recovered with a probability of 0.18 (Fig 3).

### Estimation of trait-dependent speciation rates

**Fig 4:**
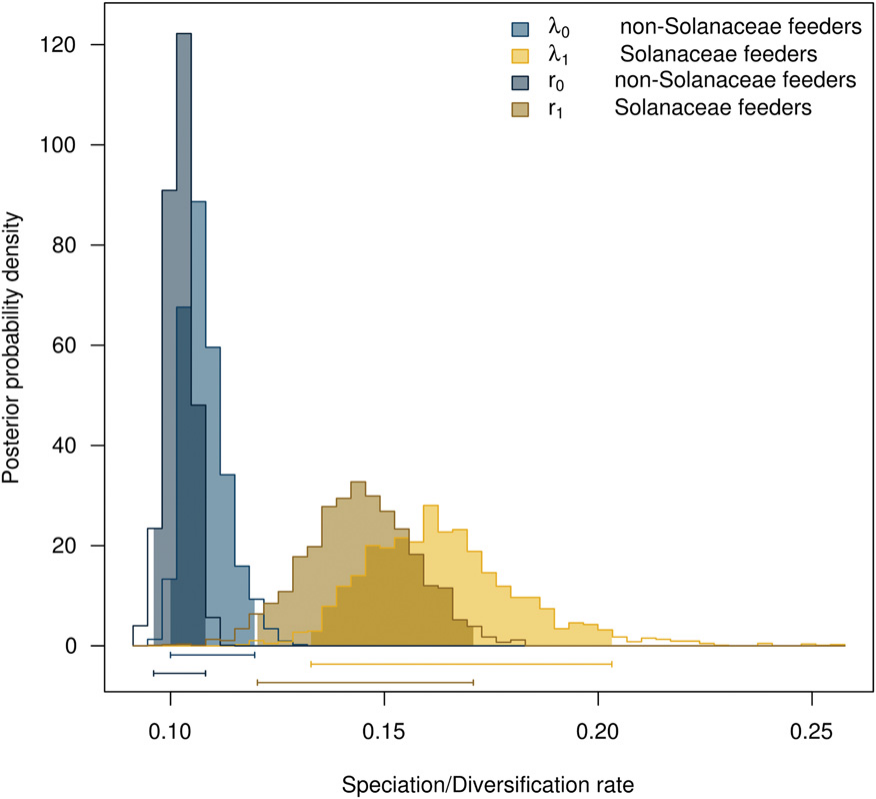
BiSSE analysis of diversification of nymphalids due to feeding on Solanaceae hostplants. Speciation and net diversification rates are significantly higher in Solanaceae feeders (speciation rate = *λ*1, net diversification rate = *r*1)

The MEDUSA analyses, taking into account phylogenetic uncertainty, estimated a net diversification rate increase in part of the clade Ithomiini across more than 95% of the trees. Our BiSSE analysis found a positive effect of the character state “feeding on Solanaceae” on the net diversification rate on part of Ithomiini (Oleriina + Ithomiina + Napeogenina + Dircennina + Godyridina) (Fig 4). The estimated mean net diversification rate for taxa that do not feed on Solanaceae was r = 0.11 while the net diversification rate for the Solanaceae feeders was r = 0.16 (see S1 Fig for a boxplot of speciation and extinction values for the 95% credibility intervals). The same analysis considering *Vanessa* and *Hypanartia* as non-Solanaceae feeders due to dubious records produced the same pattern and net diversification rates (S2 Fig). Therefore, the rest of the analyses were performed assuming these two genera as Solanaceae feeders. We constrained the BiSSE likelihood model to force equal rates of speciation for both character states in order to test whether the model of different speciation rates is a significantly better explanation for the data. A likelihood ratio test found that the model for increased net diversification rate for nymphalids feeding on Solanaceae is a significantly better explanation than this character state having no effect on diversification (*χ* ^2^ = 12.3; 1*df*; *p* < 0.001) (Table 3; character states available in S4 Dataset, code in S3 Supporting Information, and mcmc run in S4 Supporting Information). We combined the post-burnin mcmc generations from running BiSSE on 250 trees from the random sample of 1000 trees and found the same pattern as the BiSSE analysis on the maximum clade credibility tree (combined mcmc run in S5 Supporting Information; profiles plot of speciation rates in S3 Fig; boxplot of 95% credibility intervals in S4 Fig). A BiSSE analysis to test whether the trait “feeding on Apocynaceae” had any effect on increased net diversification rates found similar speciation rates for lineages feeding on Apocynaceae and other plants (S5 Fig).

**Table 3:** Likelihood ratio test between the model of increased diversification of nymphalids feeding on Solanaceae against a model forcing equal speciation rates (no effect on diversification).

Df	lnLik	AIC	ChiSq	p	
full	6	-1613.3	3238.5		
equal.lambda	5	-1619.4	3248.9	12.3	0.00045

## Discussion

### Effects of phylogenetic uncertainty on the performance of MEDUSA

The MEDUSA method has been used to infer changes in net diversification rates in a phylogenetic tree. Since its publication [17] the results of using MEDUSA on a single tree, the maximum clade credibility tree, have been used for generation of hypotheses and discussion [11, 45, 46]. However, different diversification shifts and different rates of diversification are found for certain lineages when phylogenetic uncertainty was taken into account by using MEDUSA on a random sample of trees from the posterior distribution of a Bayesian run. We found that some diversification shifts, estimated on the Nymphalidae maximum clade credibility tree, were found with very low probability in the random sample of 1000 trees from the posterior distribution (Fig 3, Table 2). We also found that, even though the analyses estimated the same diversification shifts on two or more trees, the estimated net diversification rates could vary widely (Fig 2). For example, in our Nymphalidae trees, we found that the shift for *Magneuptychia* and *Caeruleuptychia* had a rate from r = 0.5234, higher than the background net diversification rate, to almost zero. This means that observed patterns and conclusions can be completely contradictory depending on tree choice.

In this study, the effect of phylogenetic uncertainty on the inferred diversification shifts by MEDUSA is amplified because some Nymphalidae taxa appear to be strongly affected by long-branch attraction artifacts [33]. Thus, the Bayesian runs are expected to recover alternative topologies on the posterior distribution of trees, resulting in low support and posterior probability values for the nodes. For example, posterior probability values for clades in Satyrinae are very low [14]. As a result, MEDUSA inferred a net diversification rate increase for the Satyrinae (which includes Satyrini and its sister clade) in the maximum clade credibility tree, but this was recovered in the MultiMEDUSA analysis in only 13% of the random sample of trees.

If there is strong phylogenetic signal for increases or decreases in net diversification rates for a node, it is expected that these shifts would be inferred by MEDUSA in most of the posterior distribution of trees. However, weak phylogenetic signal for some nodes can cause some clades to be absent in some trees and MEDUSA will be unable to estimate any diversification shift (due to a non-existent node). The relatively low phylogenetic support for many nodes in the Nymphalidae tree is likely the reason why MEDUSA estimated net diversification rate shifts with a probability higher than 0.90 in the sample of trees for only four shifts: the genus *Charaxes*, the genus *Ypthima*, part of Ithomiini and the clade *Callicore* + *Diaethria* (Fig 3), while estimating shifts for other lineages with much lower probability.

### Hostplant use and diversification in Nymphalidae

#### Ithomiini

Keith Brown suggested that feeding on Solanaceae was an important event in the diversification of Ithomiini butterflies [47]. Ithomiini butterflies are exclusively Neotropical and most species feed on Solanaceae hostplants during larval stage [9]. Optimizations of the evolution of hostplant use on phylogenies evidence a probable shift from Apocynaceae to Solanaceae in the ancestor of the tribe [9, 48]. Fordyce (2010) [13] found that the Gamma statistics, a LTT plot of an Ithomiini phylogeny and the fit of the density-dependent model of diversification are consistent with a burst of diversification in Ithomiini following the shift from Apocynaceae to Solanaceae.

We investigated whether the strong signal for an increase in net diversification rate for Ithomiini (found by MEDUSA) can be explained due to the use of Solanaceae plants as hosts during larval stage. For this, we used a Bayesian approach [49] to test whether the trait “feeding on Solanaceae” had any effect on the diversification of the group.

Our BiSSE analysis, extended to take into account missing taxa and phylogenetic uncertainty, shows a significantly higher net diversification rate for Ithomiini taxa, which can be attributed to the trait “feeding on Solanaceae hostplants” (Fig 4). This is in agreement with previous findings using other statistical methods [13]. Due to the fact that Ithomiini are virtually the only nymphalids using Solanaceae as hostplants, it is possible that the trait responsible for a higher diversification of Ithomiini might not be the hostplant character. As noted by Maddison et al. (2007) [42], the responsible trait might be a codistributed character such as a trait related to the ability to digest secondary metabolites.

Solanaceae plants contain chemical compounds and it has been suggested that the high diversity of Ithomiini is consistent with the “escape-and-radiate scenario” due to a shift onto Solanaceae [13] and radiation scenarios among chemically different lineages of Solanaceae plants [9, 47]. According to this theory, the shift from Apocynaceae to Solanaceae allowed Ithomiini to invade newly available resources due to a possible key innovation that allowed them cope with secondary metabolites of the new hosts. Additional studies are needed to identify the actual enzymes that Ithomiini species might be using for detoxification of ingested food as they have been found in other butterfly groups [50].

The increase in diversification rate inferred by MEDUSA occurred after the probable shift from Apocynaceae to Solanaceae, as the Solanaceae feeders in the subtribes Melinaeina and Mechanitina are not included in the diversification shift (shift number 5 in Fig 1). The apparent conflicting results from MEDUSA and BiSSE can be explained by the low species-richness of the subtribes Melinaeina and Mechanitina compared to the other subtribes included in the shift (52 versus 272 species). It can be that MEDUSA is more conservative than BiSSE and is not including Melinaeina and Mechanitina in the shift due to low species numbers.

Although the Solanaceae genera used by the Ithomiini clades are well known [9], we do not have any understanding on the physiological routes involved in the detoxification of Solanaceae compounds by the several lineages of Ithomiini. We can speculate that older lineages exploiting a novel toxic resource [9, 14] may be inefficient in metabolizing plant toxins and that younger lineages are able to deal with toxins more efficiently, so that host switching events within Solanaceae are possible, which can lead to higher diversification. Studies in *Papilio* species have reported that detoxification enzymes can become more efficient in metabolizing toxins than ancestral configurations of the proteins, providing more opportunities for hostplant switches [51]. This might be the reason why the basal Ithomiini subtribes Melinaeina and Mechanitina are so species-poor and restricted to few Solanaceae hosts [9], while recent subtribes are species-rich and have expanded their host range into several Solanaceae lineages [9]. It might be that the switch to feeding in Solanaceae was an important event in the evolutionary history of Ithomiini, but the actual radiation occurred after critical physiological changes (a probable key innovation) allowed efficient detoxification of Solanaceae toxins.

The diffuse cospeciation hypothesis predicts almost identical ages of insects and their hostplants, while the “resource abundance-dependent diversity” and the “escape-and-radiate” hypotheses posit that insects diversify after their hostplants [2–4]. Wheat et al. (2007) [50] found strong evidence for a model of speciation congruent with Ehrlich and Raven’s hypothesis in Pieridae butterflies due to, in addition to the identification of a key innovation, a burst of diversification in glucosinolate-feeding taxa shortly afterwards (with a lag of circa 10 MY). According to a recent dated phylogeny of the Angiosperms [52], the family Solanaceae split from its sister group about 59 (49–68) MYA and diversification started (crown group age) around 37 (29–47) MYA. Wahlberg et al. (2009) [14] give the ages for origin and diversification for Ithomiini at 45 (39–53) and 37 (32–43) MYA, respectively. Thus, current evidence shows that Solanaceae and Ithomiini might have diversified around the same time, during the Late Eocene and Oligocene, and this would be congruent with the diffuse cospeciation hypothesis.

#### Danaini

Our MultiMEDUSA approach showed a significant slowdown in net diversification rate in the subtribe Danaina of the Danini. Both Danaina and the sister clade Euploeina feed mainly on Apocynaceae and thus a hostplant shift should not be responsible for the observed slowdown of diversification in the Danaina. As expected, our BiSSE analysis of Apocynaceae feeders shows that there is no effect of feeding on this plant family on the net diversification rates of Nymphalidae lineages. Many of the Danaina are large, strong fliers, highly migratory and involved in mimicry rings, including the best-known migratory butterfly, the monarch (*Danaus plexippus*). The causes for a lower net diversification rate in the Danaina remains to be investigated, but their great dispersal power might be involved in preventing allopatric speciation. It has been found in highly vagile species in the nymphalid genus *Vanessa* that dispersal has homogenized populations due to gene flow, as vagile species seem to be genetically homogeneous among populations [53].

#### Satyrinae

Lineages in the diverse family Satyrinae radiated simultaneously with the radiation of their main hostplant, grasses, during the climatic cooling in the Oligocene [15]. Thus, it is somewhat surprising that part of Satyrinae were found to have accelerated diversification in only 13% of the trees from the posterior distribution. Although this can be attributed to low phylogenetic signal [14], the clade Satyrini is very robust [14] and MEDUSA failed to identify any significant accelerated net diversification rate for Satyrini. It appears that the radiation of Satyrini as a whole was not remarkably fast and therefore not detected by MEDUSA, although it estimated a diversification shift for Satyrini + its sister clade. This should be expected if the diversification of Satyrini occurred in a stepwise manner, with pulses or bursts of diversification for certain lineages but unlikely for the tribe Satyrini as a whole.

## Conclusions

We found that even though MEDUSA estimated several diversification shifts in the maximum clade credibility tree of Nymphalidae, only a few of these shifts were found in more than 90% of the trees from the posterior distribution. In the literature, it is common practice that conclusions are based on the shifts estimated on the maximum clade credibility tree. However, by using a MultiMEDUSA approach, we found that for this Nymphalidae dataset some of these shifts might be greatly affected by phylogenetic uncertainty. Moreover, some of these shifts can be recovered either as increases or decreases in net diversification rate depending on the tree from the posterior distribution that was used for analysis. This means that contradictory conclusions would be made if only the maximum clade credibility tree was used for analysis. We recommend that all datasets should be analyzed using the MultiMEDUSA approach in order to test whether the results are robust when phylogenetic uncertainty is taken into account.

MEDUSA appears to be sensitive to the number of nodes with high posterior probability and width of age confidence intervals. For our data, it would be necessary to obtain a posterior distribution of trees with no conflicting topology, and very similar estimated ages for nodes in order to consistently recover most of the diversification shifts on the posterior distribution of trees that were inferred by MEDUSA on the MCC tree.

Our MultiMEDUSA approach to perform analyses on the posterior distribution of trees found strong support for an increase in net diversification rate in the tribe Ithomiini, the genus *Ypthima*, genus *Charaxes*, the clade *Callicore* + *Diaethria* and for a decrease in net diversification rate in the subtribe Danaina. Due to phylogenetic uncertainty, we did not obtain strong support for other diversification shifts in Nymphalidae. Our BiSSE analysis corroborated other studies in that Solanaceae-feeding, or a codistributed character, was likely important in the diversification of Ithomiini butterflies. However, by applying MEDUSA we found that a critical character in the radiation of the group might have appeared after the shift from Apocynaceae to Solanaceae. We also found that Apocynaceae-feeding is not responsible for the slowdown of diversification in Danaina. Ithomiini and Solanaceae diversified near simultaneously, which is in agreement with the diffuse cospeciation hypothesis [2, 4].

## Supporting Information

S1 Fig Boxplot of speciation (*λ*) and extinction (*μ*) values for the 95% credibility intervals of values estimated by BiSSE analysis of diversification due to feeding on Solanaceae plants. (PDF)

S2 Fig BiSSE analysis of diversification of nymphalids due to feeding on Solanaceae hostplants assuming *Vanessa* and *Hypanartia* as non-Solanaceae feeders. The same pattern is recovered, speciation and net diversification rates are significantly higher for Solanaceae feeders *λ*1, r1).
(PDF)

S3 Fig Net diversification rates of nymphalids feeding on Solanaceae plants as estimated by combining post-burnin runs of BiSSE on the 1000 trees from the posterior distribution. (PDF)

S4 Fig Boxplot of speciation and extinction values for the 95% credibility intervals of values estimated by BiSSE analysis of diversification due to feeding on Solanaceae plants on the combined post-burnin runs on 1000 trees from the posterior distribution. (PDF)

S5 Fig BiSSE analysis of diversification of nymphalids due to feeding on Apocynaceae hostplants. Speciation and net diversification rates are similar.
(PDF)

S6 Fig Probability of nodes with estimated rates from a MultiMEDUSA run on the 1000 random trees selected from the posterior distribution. (PDF)

S7 Fig Summary of the MultiMEDUSA analysis on the 1000 trees from the posterior distribution. (PDF)

S8 Fig Figure for MEDUSA run on MCC tree from random 1000 trees. (PDF)

S1 File 1000 random trees from [14]. (ZIP)

S2 File R script to run MEDUSA on the MCC Nymphalidae tree from [14]. This script removes the outgroup taxa and loads the richness data for the tree terminals.
(TXT)

S3 File R script to run a MultiMEDUSA analysis on 1000 random trees from [14]. (TXT)

S4 File Raw results from the MultiMEDUSA run on the random sample of trees from the posterior distribution. (ZIP)

S1 Dataset Species richness for lineages in Nymphalidae. (CSV)

S2 Dataset Hostplants of Nymphalidae butterflies recorded from the literature. (CSV)

S3 Dataset References for hostplants data. (CSV)

S4 Dataset Data matrix with character states for hosplant use. (CSV)

S5 Dataset MCC Nymphalidae tree from [14]. (TXT)

S1 Supporting Information R script to run a MEDUSA analysis on the MCC tree from the 1000 random trees selected from the posterior distribution. (TXT)

S2 Supporting Information MCC tree from the 1000 random trees selected from the posterior distribution. (CSV)

S3 Supporting Information R script for running the BiSSE analysis. (TXT)

S4 Supporting Information Raw results for the BiSSE analysis. (CSV)

S5 Supporting Information Raw results for the combined BiSSE analysis. (CSV)

S1 Text Summary results from a MultiMEDUSA run on the 1000 random trees selected from the posterior distribution. (TXT)

S2 Text Results of running MEDUSA on the MCC tree. (TXT)

S3 Text Summary results from a MEDUSA run on the MCC tree from the 1000 random trees selected from the posterior distribution. (TXT)
