# Supplementary figures and images for: Diversity Dynamics in Nymphalidae Butterflies: Effect of Phylogenetic Uncertainty on Diversification Rate Shift Estimates
Source: PLoS One. 2015 Apr 1;10(4):e0120928. doi: 10.1371/journal.pone.0120928 (PMC4382342; doi:10.1371/journal.pone.0120928)

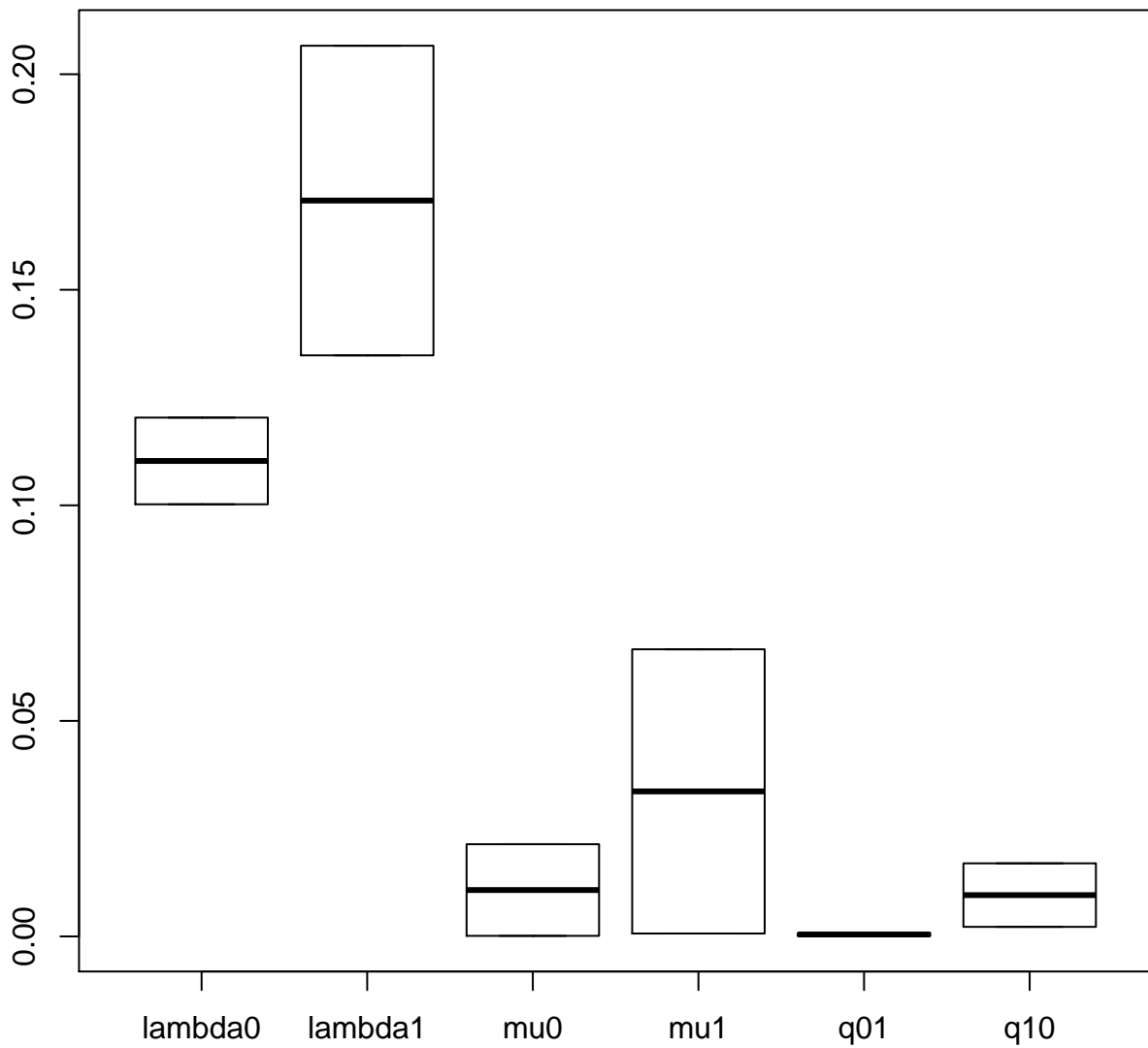

Supplement: S1 Fig — (PDF) [file pone.0120928.s001.pdf]

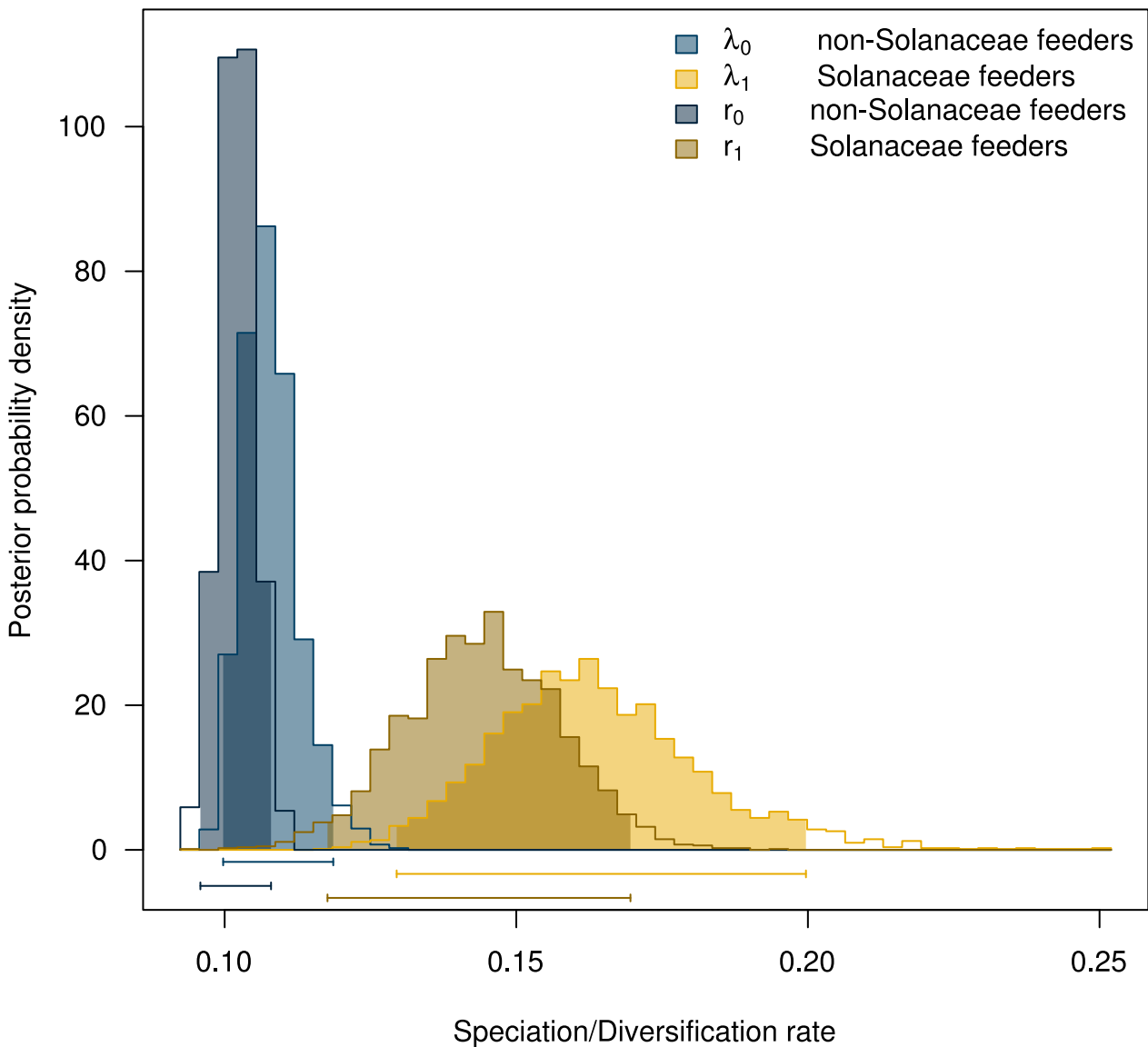

Supplement: S2 Fig — The same pattern is recovered, speciation and net diversification rates are significantly higher for Solanaceae feeders λ1, r1). (PDF) [file pone.0120928.s002.pdf]

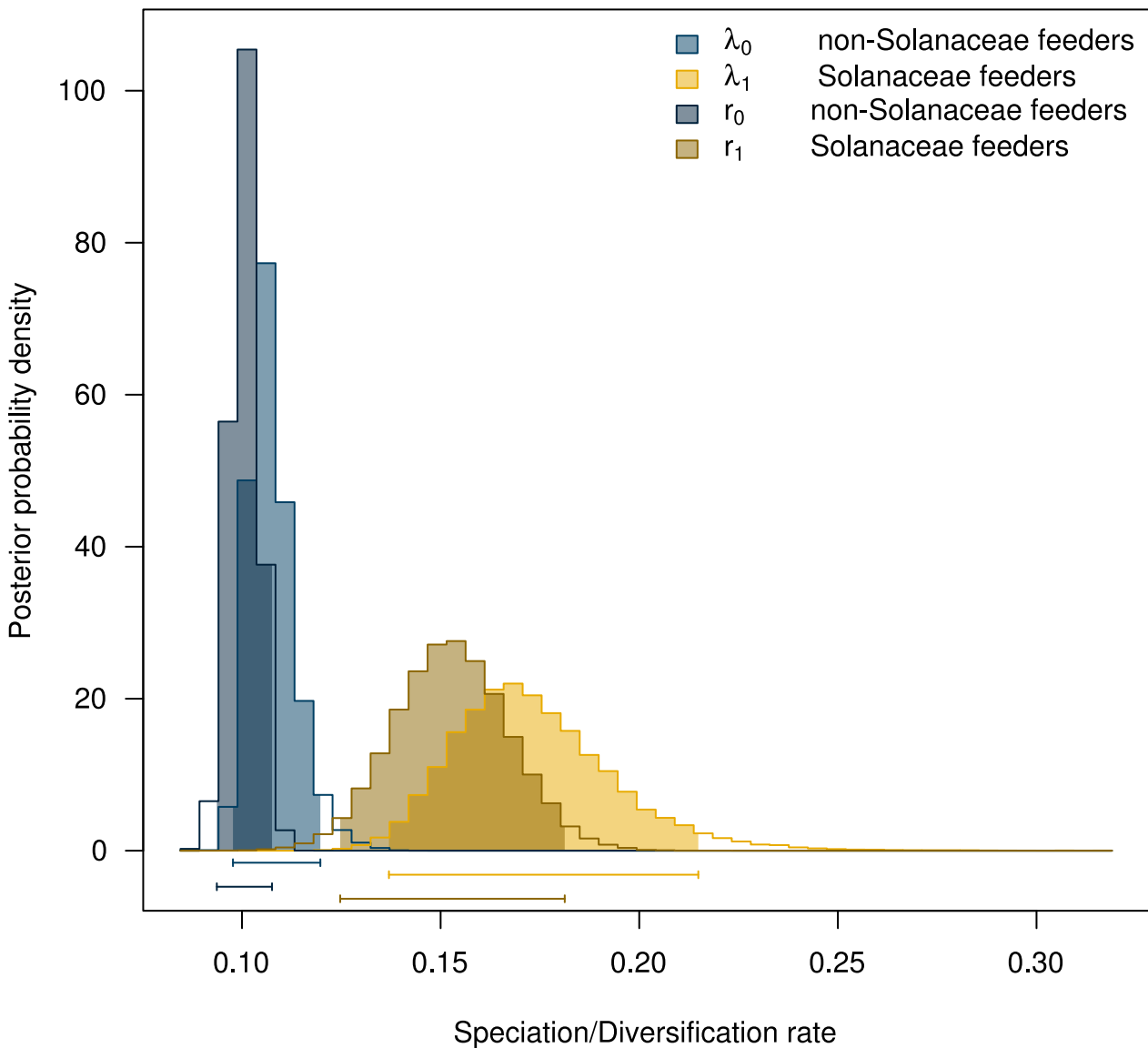

Supplement: S3 Fig — (PDF) [file pone.0120928.s003.pdf]

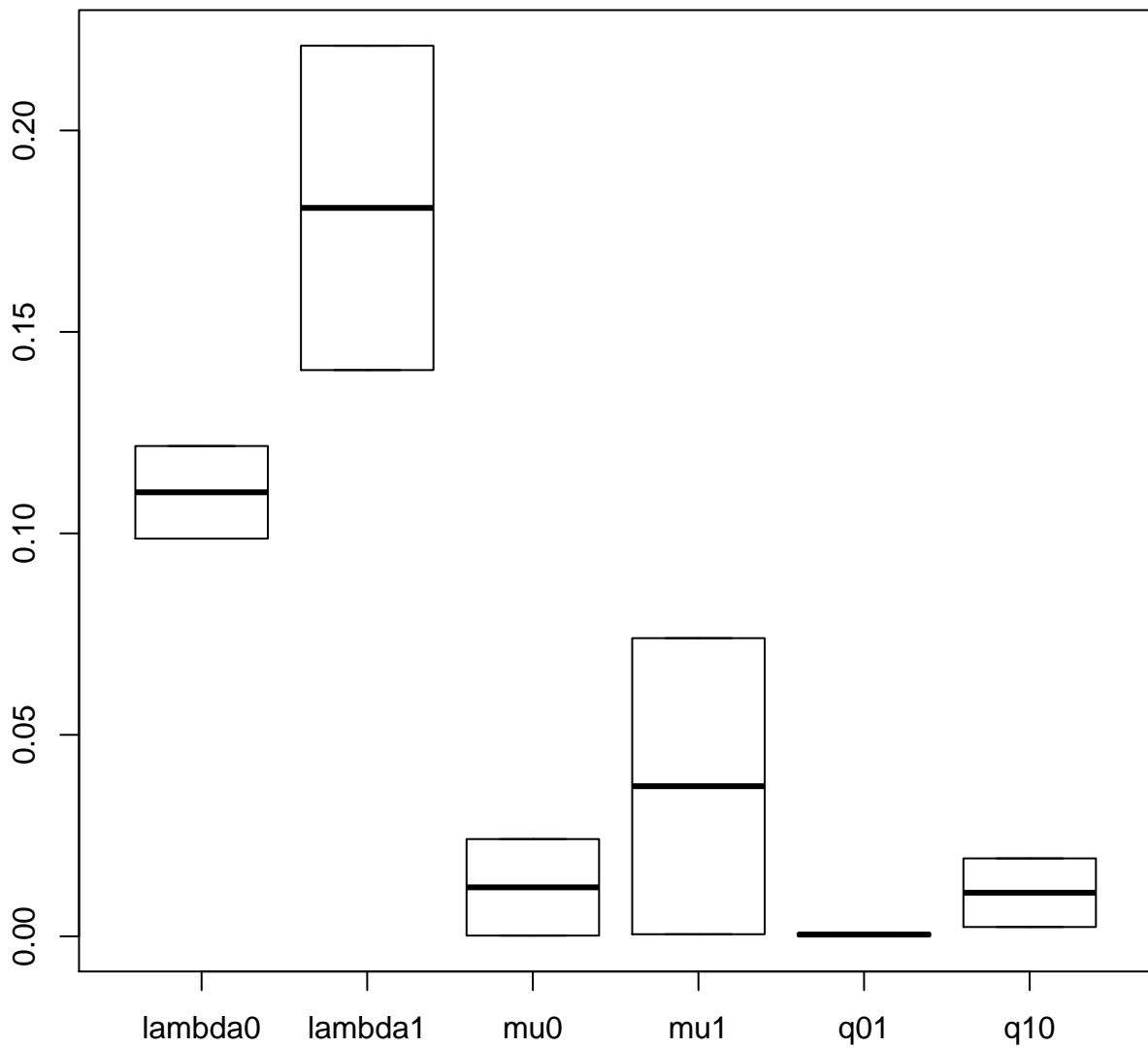

Supplement: S4 Fig — (PDF) [file pone.0120928.s004.pdf]

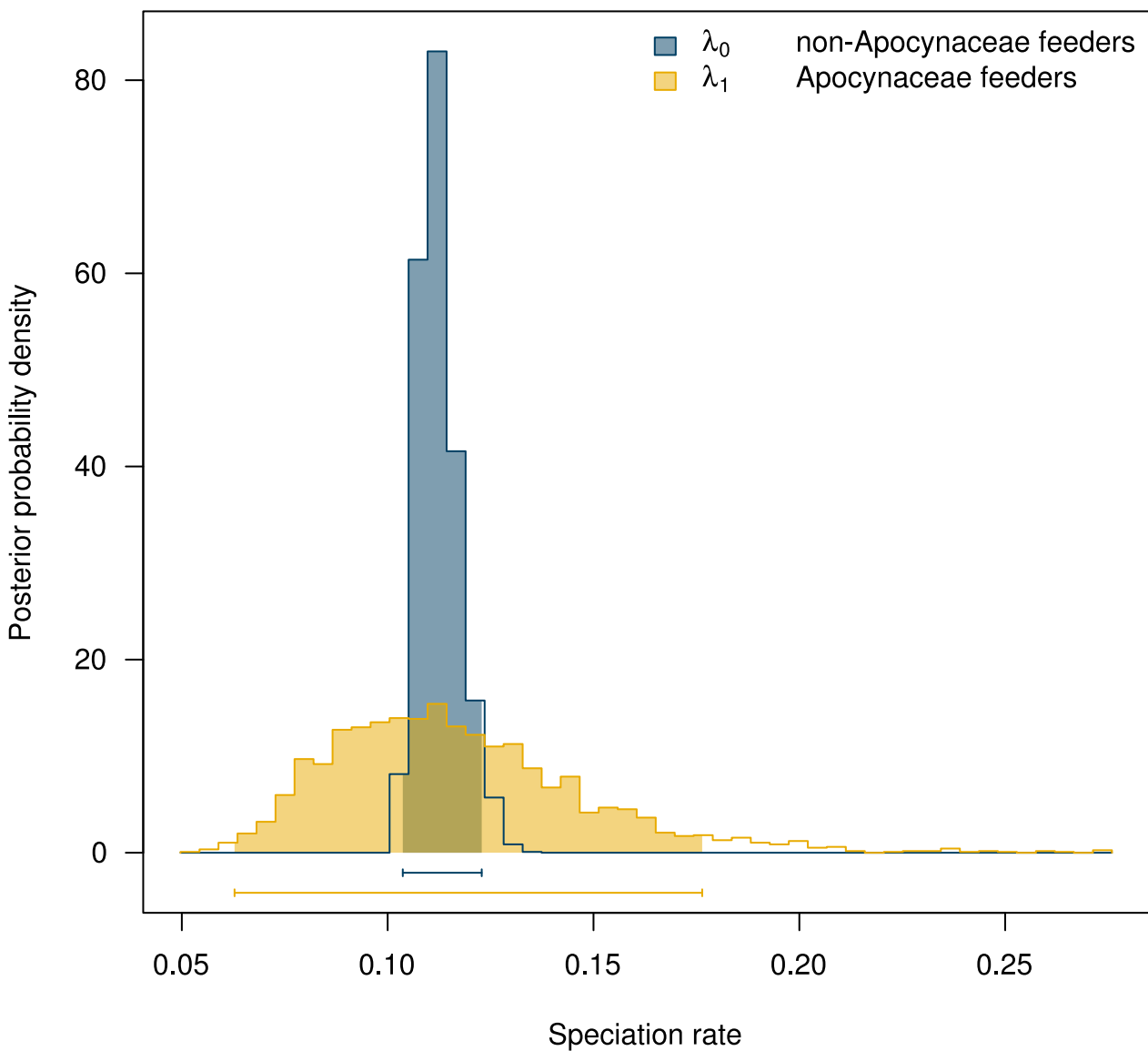

Supplement: S5 Fig — Speciation and net diversification rates are similar. (PDF) [file pone.0120928.s005.pdf]

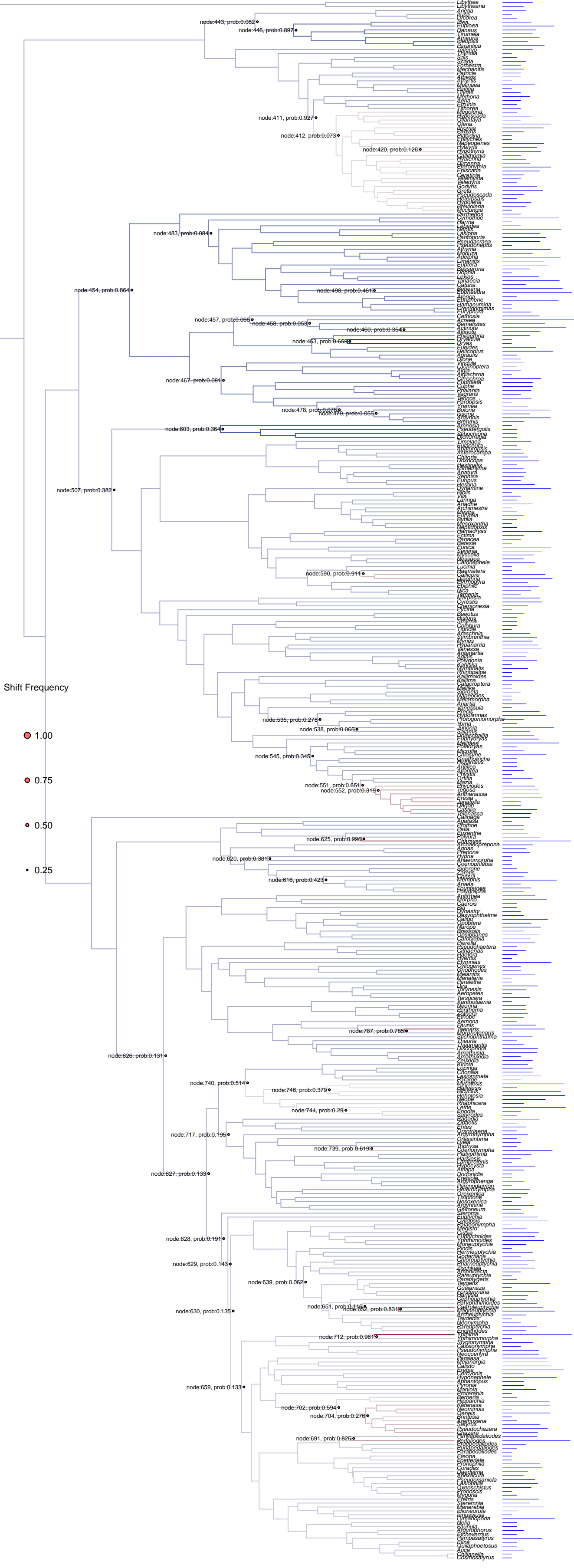

Shift Frequency

1.00

0.75

0.50

0.25

Divergence Time (MYA)

ln(species count + 1)

Supplement: S6 Fig — (PDF) [file pone.0120928.s006.pdf]

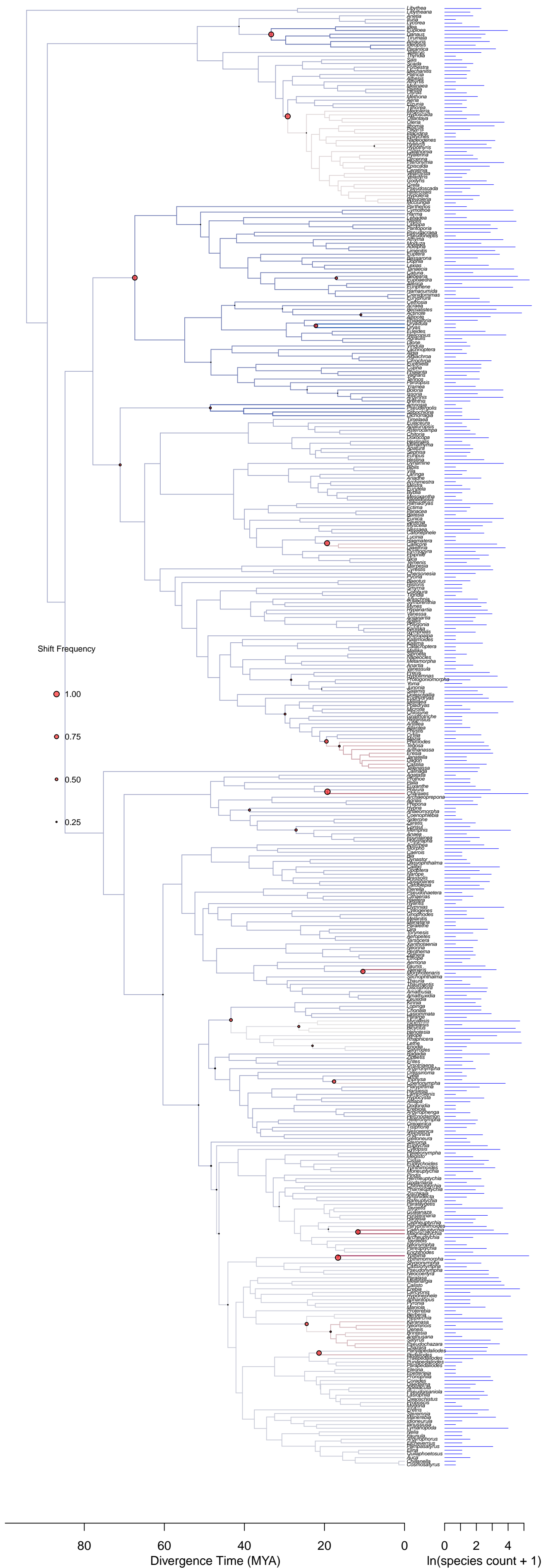

Supplement: S7 Fig — (PDF) [file pone.0120928.s007.pdf]

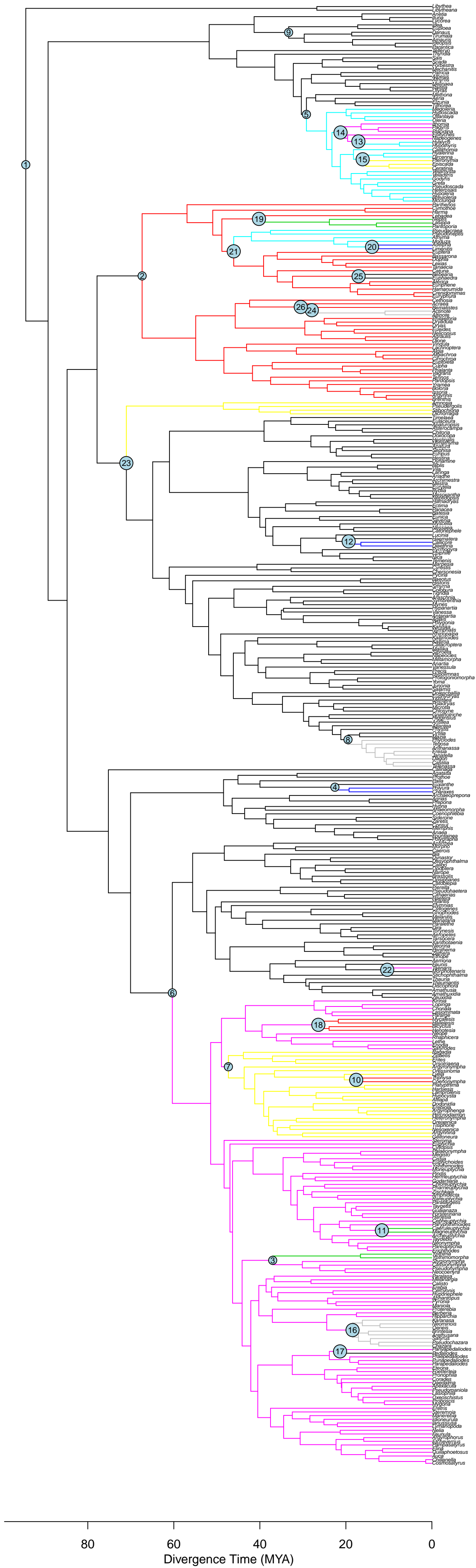

Supplement: S8 Fig — (PDF) [file pone.0120928.s008.pdf]
